# BCR signaling contributes to autophagy regulation in chronic lymphocytic leukemia

**DOI:** 10.1038/s41375-019-0557-y

**Published:** 2019-08-28

**Authors:** Lindsay D. Smith, Annabel R. Minton, Matthew D. Blunt, Laura I. Karydis, David A. Dutton, Karly-Rai Rogers-Broadway, Rachel Dobson, Rena Liu, Faith Norster, Elizabeth Hogg, Margaret Ashton-Key, Jonathan C. Strefford, Li Jia, Dimitar G. Efremov, G. Vignir Helgason, Peter W. M. Johnson, Freda K. Stevenson, Francesco Forconi, Mark S. Cragg, David A. Tumbarello, Graham Packham, Andrew J. Steele

**Affiliations:** 1 Cancer Sciences, University of Southampton, Southampton General Hospital, Somers Cancer Sciences Building, Southampton, SO16 6YD UK; 2 Haemato-Oncology, Barts Cancer Institute, Queen Mary University of London, London, EC1M 6BQ UK; 3 Department of Cellular Pathology, Southampton University Hospital Trust, Southampton, SO17 1BJ UK; 4 Molecular Hematology Unit, International Centre for Genetic Engineering & Biotechnology, Padriciano 99, 34149 Trieste, Italy; 5 Paul O’Gorman Leukaemia Research Centre, Institute of Cancer Sciences, College of Medical, Veterinary, and Life Sciences, University of Glasgow, Glasgow, G12 0ZD UK; 6 Institute for Life Sciences, University of Southampton, University Road, Highfield Campus, Southampton, SO17 1BJ UK

**Keywords:** B cells, Chronic lymphocytic leukaemia, Cell signalling

## To the Editor

Chronic lymphocytic leukemia (CLL) is characterized by the accumulation of tumor B-cells within the blood and tissues [1]. Patients with unmutated (U-CLL) or mutated (M-CLL) *IGHV* genes typically have a progressive or indolent disease, respectively. However, B-cell receptor (BCR) signaling adds a further complexity and is pivotal to CLL pathogenesis, promoting tumor survival, proliferation, and consequently tumor progression [2]. Indeed, inhibition of this pathway has revolutionized clinical responses in patients [3].

Macroautophagy (herein Autophagy) maintains cellular homeostasis through bulk protein degradation. During the autophagy process LC3 (microtubule associated protein 1 light chain 3) and GABARAP (GABA type A receptor proteins) family proteins, ATG3 and ATG7 are essential for autophagosome formation, autophagosome–lysosome fusion, and cargo degradation (Supplementary Fig. S1) [4]. In CLL, high basal expression of autophagy genes, *BECN1*, *PIK3C3*, and *PIK3R4*, have been associated with a shorter time-to-first treatment (TTFT) and worse overall survival in patients [5]. Furthermore, BECN1 and ATG5 RNA expression have been shown to correlate with poorer clinical outcome [6] and autophagy was shown to reduce therapy-induced apoptosis [7]. However, how BCR activation regulates autophagy in CLL has not been previously evaluated.

**Fig. 1:**
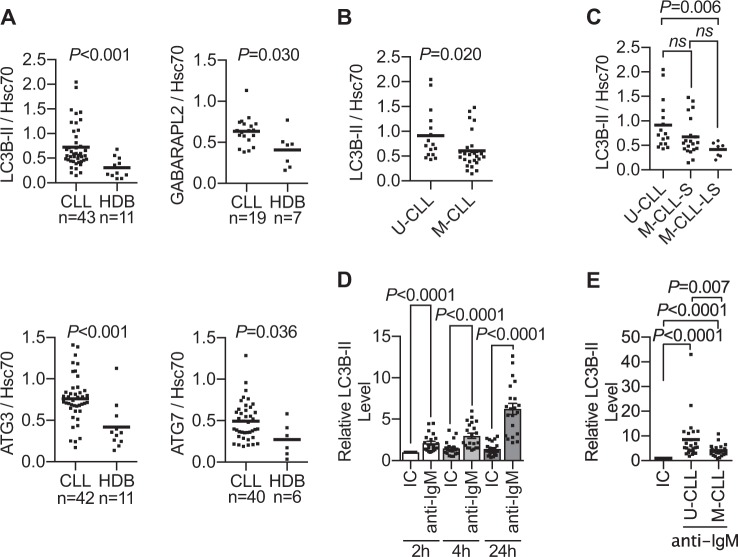
Characterization of autophagy-marker protein levels at baseline and following BCR engagement. **a** Protein was extracted from snap-frozen PBMCs isolated from CLL patients or HDB purified by negative selection. The level of LC3B-II (*n* = 43), GABARAPL2 (*n* = 19), ATG3 (*n* = 42), and ATG7 (*n* = 40) was quantified by immunoblotting and normalized protein levels relative to the Hsc70 loading control are shown. Mean values are indicated. A Mann–Whitney test was used for statistical analysis. **b** Basal LC3B-II protein levels in CLL samples (*n* = 43) divided by *IGHV* mutational status into mutated (M-CLL) and unmutated (U-CLL) cases, and (**c**) BCR signaling capacity defined by U-CLL, M-CLL signallers (M-CLL-S) (>5% iCa^2+^ flux), and M-CLL low signallers (M-CLL-LS) (≤5% iCa^2+^ flux). Mean values are indicated. A Mann–Whitney test was used for statistical analysis. **d** CLL samples were treated with bead-bound isotype control antibody (IC), anti-IgM, or anti-IgD for 2, 4, or 24 h, and the level of LC3B-II (*n* = 21) assessed by immunoblotting. Blots were quantified and the mean fold change (±SEM) in the level of LC3B-II with anti-IgM normalized to the IC at 2 h is shown. A Wilcoxon’s matched-pairs signed-rank test was  used for statistical analysis. **e** A larger cohort of patient samples (*n* = 45) were treated with bead-bound anti-IgM for 24 h and LC3B-II levels divided according to *IGHV* status into M-CLL and U-CLL as previously described. Mean values are indicated. A Mann–Whitney test was used for statistical analysis

Evaluation of basal autophagy-protein levels demonstrated significantly greater LC3B-II, GABARAPL2, ATG3, and ATG7 in CLL cases (Supplementary Table S1; for antibody information, see Supplementary Table S2) compared with healthy donor B-cells (HDB) (Fig. 1a and Supplementary Fig. S2A). The basal levels of LC3B-II, but not GABARAPL2, ATG3, or ATG7, were significantly greater still in U-CLL compared with M-CLL (Fig. 1b and Supplementary Fig. S2B). In addition, LC3B-II and ATG3 levels, but not GABARAPL2 or ATG7, associated with BCR signaling capacity (Fig. 1c and Supplementary Fig. S2C). With significantly greater levels of LC3B-II and ATG3 observed within U-CLL compared with MCLL-LS due to the contrasting extremes of anti-IgM-mediated signaling between these two subgroups. M-CLL-S samples that retained anti-IgM signaling capacity expressed LC3B-II at an intermediate level between U-CLL and M-CLL-LS cases (Fig. 1c), suggesting LC3B-II levels were influenced by both inherent BCR signaling and the cells’ origin. Indeed, greater LC3B-II levels were associated with a significantly shorter TTFT from the first lymphocytosis (Supplementary Fig. S3A**)** irrespective of *IGHV* mutational status, as both cohorts expressed a mixture of U-CLL and M-CLL cases. Intriguingly, although numbers were small, LC3B-II expression also appeared to identify M-CLL patients, but not U-CLL patients, which required earlier treatment (Supplementary Fig. S3B). However, further work in a larger trial using a fully quantitative technique is required to identify the potential of LC3B-II as a biomarker.

To determine whether autophagy occurred in vivo, we performed immunohistochemistry on lymph node tissue from CLL patients and control tissue from healthy, reactive nodes. LC3B expression was detected most strongly within proliferation centers from sequential tissue sections labeled with Ki67 (Supplementary Fig. S4A, B) and in reactive node germinal centers (Supplementary Fig. S4D). This shows that LC3B expression is associated with CLL cells and B-cells that had undergone antigen engagement. These cells appeared to be B-cells given that the majority of cells were CD20+ in sequential tissue sections (Supplementary Fig. S4C, D).

Consequently, we assessed whether BCR engagement for 2, 4, or 24 h with bead-bound anti-IgM or anti-IgD could modify expression of key autophagy proteins LC3B-II, GABARAPL2, ATG3, and ATG7 in CLL cells **(**Fig. 1d and Supplementary Fig. S5A, B). Significant increases in the level of all proteins were observed 24 h following treatment with anti-IgM. However, significant increases in GABARAPL2 and LC3B-II also occurred earlier between 2 and 4 h, whereas  ATG3 and ATG7 only demonstrated trends for increased expression at this time. Subsequent evaluation of LC3B-II expression in a larger cohort of patients treated with bead-bound anti-IgM for 24 h demonstrated that the majority of CLL cases activated their autophagy pathway in response to BCR engagement (Supplementary Fig. S5C). Furthermore, U-CLL cases expressed greater levels of LC3B-II in response to anti-IgM compared with M-CLL cases (median fold increase of 5.4 and 3.5, respectively; Fig. 1e). Some overlap in the anti-IgM-mediated LC3B-II levels was identified between *IGHV* subsets that maybe a result of M-CLL cases with active BCR signaling. Compared with anti-IgM, anti-IgD-induced signaling is short-lived in CLL cases [8]. Therefore, although significant increases in LC3B-II were observed following bead-bound anti-IgD treatment at all time points, with trends for increased GABARAPL2, in most instances these increases were smaller than that observed with anti-IgM (Supplementary Fig. S5D). Similarly, a small but significant increase in ATG7 levels were observed at 24 h with bead-bound anti-IgD, with only a trend for increased ATG3 expression at 24 h (Supplementary Fig. S5D).

We subsequently characterized the signaling pathways involved in BCR-mediated autophagy (Supplementary Fig. S6). Phospho-ERK and -AKT levels increased 15 min post stimulation with bead-bound anti-IgM or anti-IgD, which is indicative of active BCR signaling. Interestingly, anti-IgM and anti-IgD increased phospho-p70 S6 kinase levels at 6 h, indicating active mammalian target of rapamycin signaling, despite an induction of LC3B-II expression, suggesting activation of non-canonical autophagy [9]. At 24 h, anti-IgM- and anti-IgD-dependent increases in LC3B-II, p62, and phospho-ATG13 suggested simultaneous activation of canonical and non-canonical autophagy [9, 10].

Next, we assessed autophagosome formation using LC3B puncta formation by immunofluorescence [11]. Basal autophagy was observed in cells treated with bead-bound control antibody (Supplementary Fig. S7A, B), whereas treatment with bead-bound anti-IgM increased LC3B puncta to levels above that seen in the control (Supplementary Fig. S7A, B).

To confirm that bead-bound anti-IgM-mediated autophagy was BCR-mediated and not a bead-dependent affect, CLL cells were treated with bead-bound or soluble anti-IgM in the presence or absence of the autophagosome inhibitor hydroxychloroquine (HCQ). Both soluble and bead-bound anti-IgM significantly increased LC3B-II and GABARAPL2 levels compared with the control (Supplementary Fig. S8A, B), confirming that the induction of autophagy proteins was mediated by BCR engagement and was not a bead-dependent effect. Treatment with soluble anti-IgM resulted in smaller autophagy protein increases in the same CLL samples compared with bead-bound anti-IgM, likely due to the relatively higher BCR signal strength and duration with bead-bound verse soluble anti-IgM [12].

To determine whether the anti-Ig-dependent regulation of autophagy was B-cell or tumor specific, we treated HDB with bead-bound or soluble anti-IgM or anti-IgD for 24 h. Bead-bound anti-IgM, and to a lesser extent anti-IgD, promoted significant increases in LC3B-II levels (Supplementary Fig. S9A, B), suggesting that this phenomenon was B-cell and not tumor-specific. However, in contrast to CLL, these changes were not consistently replicated in the other autophagy markers examined.

**Fig. 2:**
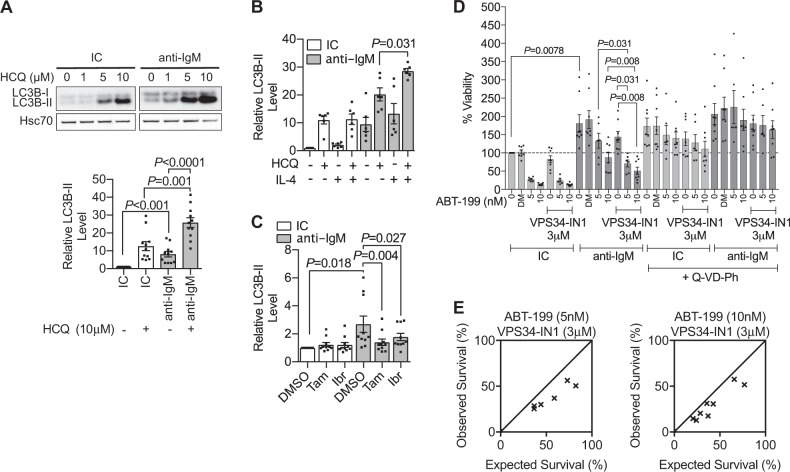
BCR-mediated autophagy is dependent on BCR signaling and provides a survival advantage to CLL cells. **a** CLL samples (*n* = 11) were treated with bead-bound isotype control antibody (IC) or anti-IgM with or without HCQ at indicated concentrations for 24 h and LC3B-II levels evaluated by immunoblotting. A representative immunoblot is shown. Blots were quantified and the mean fold change (±SEM) in LC3B-II level with each treatment vs. IC without HCQ is shown in the accompanying graph. A *t*-test was used for statistical analysis. **b** CLL samples (*n* = 6) were treated with or without IL-4 (10 ng/ml), in the presence or absence of HCQ, for 24 h before treatment with bead-bound IC or anti-IgM for 24 h. LC3B-II levels were evaluated by immunoblotting and the mean fold change (±SEM) in LC3B-II levels with each treatment vs. IC without HCQ is shown. A Wilcoxon’s matched-pairs signed-rank test was used for statistical analysis. **c** CLL samples (*n* = 10) were treated with HCQ and a SYK (tamatinib; Tam) or BTK (ibrutinib; Ibr) inhibitor (both 5 μM) for 1 h before stimulation with bead-bound IC or anti-IgM for 24 h and the LC3B-II level evaluated by immunoblotting. Hsc70 was used as a loading control. A representative immunoblot is shown. Blots were quantified and the mean fold change (±SEM) in LC3B-II level with each treatment vs. IC DMSO is shown in the accompanying graph. A Wilcoxon’s matched-pairs signed-rank test was used for statistical analysis. **d** CLL samples were treated for 6 h with bead-bound IC or anti-IgM before treatment with autophagy inhibitor, VPS34-IN1 (3 μM), either alone or in combination with venetoclax (5 nM *n* = 6, or 10 nM *n* = 8 where shown) for 24 h. All conditions were carried out with or without Q-VD-Ph (10 μM) to identify caspase-dependent drug-mediated cell killing. Cell viability was assessed by the CellTiter-Glo Cell Viability Assay. The mean (+SEM) percentage of viable cells relative to IC is shown. A Wilcoxon’s matched-pairs signed-rank test was used for statistical analysis. **e** Synergy between VPS34-IN1 (3 μM) and venetoclax (5 nm, left and 10 nM, right) was evaluated as detailed in the Supplementary Materials and Methods. XY line, observed survival = expected survival. Points below the line, synergistic interactions; points above the line, additive interactions

To determine whether BCR engagement blocked LC3B-II degradation or induced autophagosome formation, we assessed autophagic flux in response to bead-bound anti-IgM using immunoblotting in the presence or absence of HCQ as described in the autophagy guidelines [11]. CLL cells treated with HCQ accumulated LC3B-II in a concentration-dependent manner, which was further augmented with bead-bound anti-IgM (Fig. 2a). The LC3B-II increases were not a result of HCQ-mediated effects on BCR signaling (Supplementary Fig. S10) and also occurred in a time-dependent manner at the RNA level (Supplementary Fig. S11A). Comparatively, bead-bound anti-IgD stimulation produced similar but much smaller responses than anti-IgM (Supplementary Fig. S11B). These data confirm that BCR engagement does not block autosomal degradation but induces the expression of autophagy-associated genes leading to increased autophagic flux.

Next, we used interleukin (IL)-4 and BCR kinase inhibitors to confirm the role of BCR signaling in the regulation of BCR-mediated autophagy in CLL. We previously demonstrated that IL-4 induced surface IgM (sIgM) expression and subsequent downstream signaling [13]. IL-4 treatment significantly increased sIgM expression as previously demonstrated and augmented anti-IgM-dependent LC3B-II levels (Fig. 2b and Supplementary Fig. S12A, B). These affects appeared to be BCR-mediated, as IL-4 had no substantive effect on LC3B-II levels alone, even in the presence of HCQ. Next, we inhibited BCR signaling with tamatinib (SYK) and Ibrutinib (BTK), and observed the effect on LC3B-II levels. Both inhibitors significantly reduced LC3B-II to basal levels (Fig. 2c and Supplementary Fig. S12C). These data confirm the role of BCR signaling in the regulation of autophagy in CLL.

Previous studies have shown a role for autophagy in resistance to venetoclax-mediated killing in follicular lymphoma [14]. Therefore, we hypothesized that autophagy inhibitors may synergize with established therapies to inhibit basal, BCR-induced, or therapy-induced autophagy in CLL. To address this, we examined the effect on CLL cell viability of combining the autophagy inhibitor, VPS34-IN1, with venetoclax following bead-bound anti-IgM treatment. We observed significant synergy between VPS34-IN1 and venetoclax compared with single-agent treatment (Fig. 2d, e). Importantly, VPS34-IN1 inhibited BCR-mediated LC3B-II increases but had no effect on BCR signaling (Supplementary Fig. S13A, B), and both ABT-199 and VPS34-IN1-mediated cell death was largely caspase-dependent (Fig. 2d). These data indicate a protective cellular effect of BCR-mediated autophagy in CLL and highlight the therapeutic potential of inhibiting autophagy pathways to promote greater CLL cell killing.

## Supplementary information

Supplemental Material
