# Supplementary material for: BCR signaling contributes to autophagy regulation in chronic lymphocytic leukemia
Source: Leukemia. 2019 Aug 28;34(2):640–4. doi: 10.1038/s41375-019-0557-y (PMC6995694; doi:10.1038/s41375-019-0557-y)
Supplement: Supplementary file 1 — Supplemental Material [file 41375_2019_557_MOESM1_ESM.pdf]

Materials and Methods

Patient Samples

CLL diagnosis was in accordance with the IWCLL-NCI 2008 criteria.[1] Peripheral blood mononuclear cells (PBMCs) from CLL cases were studied following informed written consent in accordance with local ethics committee approvals under the declaration of Helsinki (reference 228/02/t). PBMC isolation and culture was performed as previously described.[2] HDB were isolated using a MACS B-cell isolation kit II (Miltenyi Biotec, UK). BCR signaling capacity was quantified by intracellular calcium release (iCa^{2+} flux) following soluble anti-IgM stimulation as previously described.[2, 3] The percentage of anti-IgM responding cells in each sample was calculated and a cut off of ≤5% used to distinguish between anti-IgM low and high signalers.[2] The median sIgM signaling capacity (iCa^{2+} flux) was 47% and 29% within the U-CLL and M-CLL subgroups respectively, and 47%, 54% and 3% within U-CLL, M-CLL-S and M-CLL-LS respectively.

CLL Functional Assays

PBMCs ($1x10^7$/ml) were treated with soluble (20 µg/ml) or bead-bound (2:1 bead:CLL cell ratio) anti-IgM, anti-IgD or isotype control anti-Ig goat F(ab')2 (SouthernBiotech, UK) for 24 hours unless otherwise indicated. The bead conjugation kit for the synthesis of bead-bound anti-Ig F(ab')2 was from ThermoFisher Scientific (UK). Where shown, PBMCs were pre-treated for 1 hour with VPS34IN1, venetoclax, ibrutinib or tamatinib (Stratech, UK) before anti-IgM stimulation for a further 24 hours. Hydroxychloroquine (HCQ) (10µM unless otherwise stated) (Sigma-Aldrich, UK) was added where indicated. Where shown, PBMCs were pre-treated with IL-4 (R&D Systems, UK) for 24 hours before anti-IgM stimulation for a further 24 hours. For incubations >30 minutes the caspase inhibitor Q-VD-Ph (10µM; Merck Millipore, UK) was added to minimize apoptosis.

Immunoblotting

Immunoblotting was performed as previously described.[4, 5] Membranes were probed with primary antibodies (**Supplementary Table S2**) and secondary antibodies (DAKO, UK). Proteins were visualised using a chemiDoc-it imaging system (UVP, UK). Protein densitometry was quantitated using image J (NIH, USA).

RT-PCR

Total RNA was isolated with the RNeasy mini kit (Qiagen, UK) and reverse-transcribed using the M-MLV enzyme (Promega, UK). Real-time PCR was performed using TaqMan Gene Expression Assays (Life Technologies, USA) on a 7500 Realtime PCR System (Applied Biosystems, USA). Each sample was analysed in triplicate. β2 microglobulin was used to normalise gene expression. Relative gene expression was calculated by the $2^{-\Delta\Delta CT}$ method.

Immunohistochemistry

Immunohistochemistry analysis was performed using formalin-fixed and paraffin-embedded lymph node tissue sections obtained from 30 cases of CLL. Staining was performed as detailed previously[6]. Immunostaining was performed using antibodies to LC3B (1:5000; Sigma-Aldrich, UK) and $Ki67^+$ (1:3000; DAKO, UK) via a Dako autostainer. Antibody reactions were visualised using the Super Sensitive Polymer-Horseradish Peroxidase IHC Detection System (BioGenex, USA).

Confocal Microscopy

Cells were fixed (4% PFA, 10min, RT), permeabilised (0.25% Triton, 10min, RT) and incubated with rabbit anti-LC3B (2ug/ml; Sigma L7543), followed by the secondary antibody goat anti-rabbit:AlexaFluor488 (Life Technologies) before counterstaining with DAPI (Invitrogen). Cells were washed with PBS and cytospun onto glass slides (Thermo Shandon Cytocentrifuge, C4) prior to mounting in Vectashield Hardset (Vector Labs). Images were collected on a Leica SP5 CLSM with

100x (NA1.4) Plan-apochromatic objective using LAS-AF software (Version2, Leica) and processed using Adobe Photoshop (CS6).

Flow Cytometry
CLL cells (1x10^{7}/ml) were stained for 30 minutes at 4°C with fluorescently-labeled antibodies (**Supplemental Table S2**) and the fluorescent signal quantified using a Canto II flow cytometer (BD Biosciences, UK). Data analysis was performed using FlowJo (Flowjo LLC, USA).

Cell Viability Assay
CLL cells (0.5x10^{7}/ml) were incubated with 3µm latex beads (Polysciences, USA) conjugated to isotype control antibody or anti-IgM for 6 hours before treatment with venetoclax or VPS34-IN1 either alone or combined for a further 24 hours. Cell viability was assessed using the CellTiter-Glo Cell Viability Assay (Promega, USA) as per manufacturer instructions. Additive and synergistic drug interactions were assessed as previously described[7, 8]. In brief, expected survival [(VPS34-IN1 × ABT-199)/100] was plotted against observed survival. XY line indicates observed survival = expected survival. Points beneath the line indicate synergistic interactions whereby observed survival < expected survival. Samples above the line indicate additive interactions whereby observed survival < expected survival, but observed survival is > than the most active drug used as a single-agent.

Statistical Analysis
Statistical analysis was performed using Graphpad Prism software v7 (GraphPad Software Inc., USA). Distribution of data was determined using the D'Agostino-pearson test. Statistical differences between normally distributed groups were determined by Student's t-test (paired) or Wilcoxon test (unpaired), and in all other cases by Mann-Whitney U test (unpaired). All data shown is mean ± standard error of the mean (S.E.M). P-values less than 0.05 were considered as statistically significant and only shown on graphs where significant. Survival analysis was performed using the Kaplan-Meier algorithm in SPSS. The High and Low LC3B-II cut-off was determined by ROC analysis. A Log Rank (Mantel-Cox) test was used to determine significance.

Table S1. Samples used in basal protein expression analysis.

Sample[a]	CLL (% lymphocytes)[b]	CD38 (% +ve)[c]	ZAP70 (% +ve)[c]	sIgM expression[d]	sIgD expression[d]	IgM-induced Ca2+ flux (% responding)[e]	IgD-induced Ca2+ flux (% responding)[e]	*IGHV* mutation[f]	*IGHV* usage[g]	FISH[h]
457	65	12	7	41	18	5	5	M-IGHV	V3-30	NT
488	69	4	3	22	12	15	22	M-IGHV	V1-69	No FISH lesions
520B	95	2	74	31	31	36	66	U-IGHV	V3-33	Del13q only
530B	97	87	6	115	56	74	67	U-IGHV	V3-66	Del13q only
531A	97	0	17	40	40	43	57	U-IGHV	V4-39	Del17p/TP53del
550B	89	1	2	130	69	87	91	U-IGHV	V4-34	NT
553	34	3	65	26	62	19	88	U-IGHV	V3-33	Trisomy12
555	99	25	2	38	16	68	42	U-IGHV	V3-11	Del11q/ATM
561	95	2	24	39	13	50	63	M-IGHV	V3-48	Del13q only
569	75	2	UNOB	82	17	39	64	U-IGHV	V3-7	Del13q; Del 11q/ATM
573A	87	2	3	51	59	75	81	M-IGHV	V1-3	Del13q only
575A	91	0	0	67	93	64	83	M-IGHV	V3-15	Del13q only
581	49	19	0	27	13	16	89	M-IGHV	V1-3	Del13q only
588A	94	7	5	30	94	5	63	M-IGHV	V3-23	Del13q only
589	96	0	1	43	48	71	83	M-IGHV	V1-18	Del13q only
602A	97	13	0	76	16	44	41	U-IGHV	V5-51	Del13q only
604A	92	0	2	94	58	44	66	M-IGHV	V3-30	Del13q only
609	88	18	0	25	35	57	80	M-IGHV	V3-72	Del13q only
615	93	8	5	32	11	69	59	M-IGHV	V4-39	Del13q only
618	85	33	65	24	45	58	66	U-IGHV	V3-11	No FISH lesions
620	46	1	0	39	50	9	79	M-IGHV	V2-5	NT
626	68	1	1	25	61	29	90	M-IGHV	V4-34	No FISH lesions
627	68	0	1	146	9	86	72	M-IGHV	V3-30	Del13q only
628	94	13	16	32	38	22	77	U-IGHV	V1-69	Del11q/ATM
632A	97	82	52	66	28	59	56	U-IGHV	V2-26	Del11q/ATM
633	82	0	0	45	36	59	73	M-IGHV	V3-7	Del13q only
636	94	0	0	24	7	2	2	M-IGHV	V4-34	Del11q/ATM
641	80	0	9	139	51	37	70	M-IGHV	V3-15	No FISH lesions
643	91	0	0	25	151	26	84	M-IGHV	V6-1	Del13q only
644	90	26	46	48	33	31	45	U-IGHV	V4-39	Del11q/ATM
644A	93	36	55	51	59	47	60	U-IGHV	V4-39	Del11q/ATM
645	96	0	0	43	48	66	81	M-IGHV	V1-18	NT
650	79	0	30	45	91	10	54	M-IGHV	V3-72	Del13q only
653	67	0	1	28	15	3	3	M-IGHV	V3-7	Del13q only
657B	83	0	0	18	18	2	95	M-IGHV	V4-59	Del13q only
664	93	9	24	37	10	84	63	M-IGHV	V3-21	Del11q/ATM
665	85	72	0	468	204	68	74	M-IGHV	V3-11	Trisomy12
675	88	13	8	70	6	53	12	U-IGHV	V3-21	Del17p/TP53del
684B	85	10	2	28	25	14	54	M-IGHV	V3-15	Del13q only
690	93	0	10	18	77	1	68	M-IGHV	V4-61	Del13q only
715	92	77	4	145	48	73	74	U-IGHV	V1-2	Del11q/ATM
720	92	50	67	63	70	11	79	U-IGHV	V1-69	No FISH lesions
722	77	1	0	21	16	5	49	M-IGHV	V5-51	Del13q only
732	91	47	13	85	78	47	80	U-IGHV	V1-46	Del17p/TP53del

Abbreviation: UNOB, unobtainable. NT, Not Tested.

[a] Where suffix is not shown, this is the first sample obtained from that patient, typically obtained shortly after diagnosis. A, B, C etc indicate subsequent samples. [b] Percentage of CD5+CD19+ cells. [c] Percentage of CD38+ or ZAP70+ cells. [d] Mean Fluorescence intensity (MFI) of Ig positive cells. [e] Maximal percentage of cells with increased intracellular calcium following treatment with soluble anti-Ig. [f] *IGHV* mutation status. M-IGHV, mutated; U-IGHV, unmutated. [g] IGHV gene usage. [h] Chromosomal abnormalities detected by fluorescence in situ hybridization (FISH).

IGHV gene usage and mutation status, expression of sIgM, sIgD, sCD5, sCD19 (geometric mean), CD38 and ZAP70 (% expression), and sIgM/sIgD signaling capacity (calcium flux) were determined as previously described. [2, 3]

Table S2. Antibodies used in the study.

Target	Technique	Company	Catalogue number
LC3B	Immunoblotting	Cell Signaling Technologies	3868
GABARAPL2	Immunoblotting	Cell Signaling Technologies	14256
ATG3	Immunoblotting	Cell Signaling Technologies	3415
ATG7	Immunoblotting	Cell Signaling Technologies	2631
ERK1/2	Immunoblotting	Cell Signaling Technologies	9102
AKT	Immunoblotting	Cell Signaling Technologies	9272
p70 S6 Kinase	Immunoblotting	Cell Signaling Technologies	9202
phospho-p44/42 ERK1/2 (Thr202/Tyr204)	Immunoblotting	Cell Signaling Technologies	9101
phospho-AKT (Ser473)	Immunoblotting	Cell Signaling Technologies	4060
phospho-p70 S6 Kinase (Thr389)	Immunoblotting	Cell Signaling Technologies	9205
phospho-STAT6 (Tyr641)	Immunoblotting	Cell Signaling Technologies	56554
SQSTM1/p62	Immunoblotting	Cell Signaling Technologies	5114
BCL2	Immunoblotting	DAKO	2017-06
phospho-ATG13 (Ser318)	Immunoblotting	Rockland UK	600-401-C49S
ATG13	Immunoblotting	Sigma	SAB4200100
Hsc70	Immunoblotting	Santa Cruz Biotechnology	Sc-7298
βactin	Immunoblotting	Sigma	A2228
LC3B	Immunohistochemistry	Sigma	L7543
Ki67	Immunohistochemistry	DAKO	M7240
CD20	Immunohistochemistry		L26 Clone
LC3B	Immunofluorescence	Sigma	L7543
sIgM	Flow Cytometry	DAKO	F0058
CD5	Flow Cytometry	Biolegend	300620
CD19	Flow Cytometry	Biolegend	302212

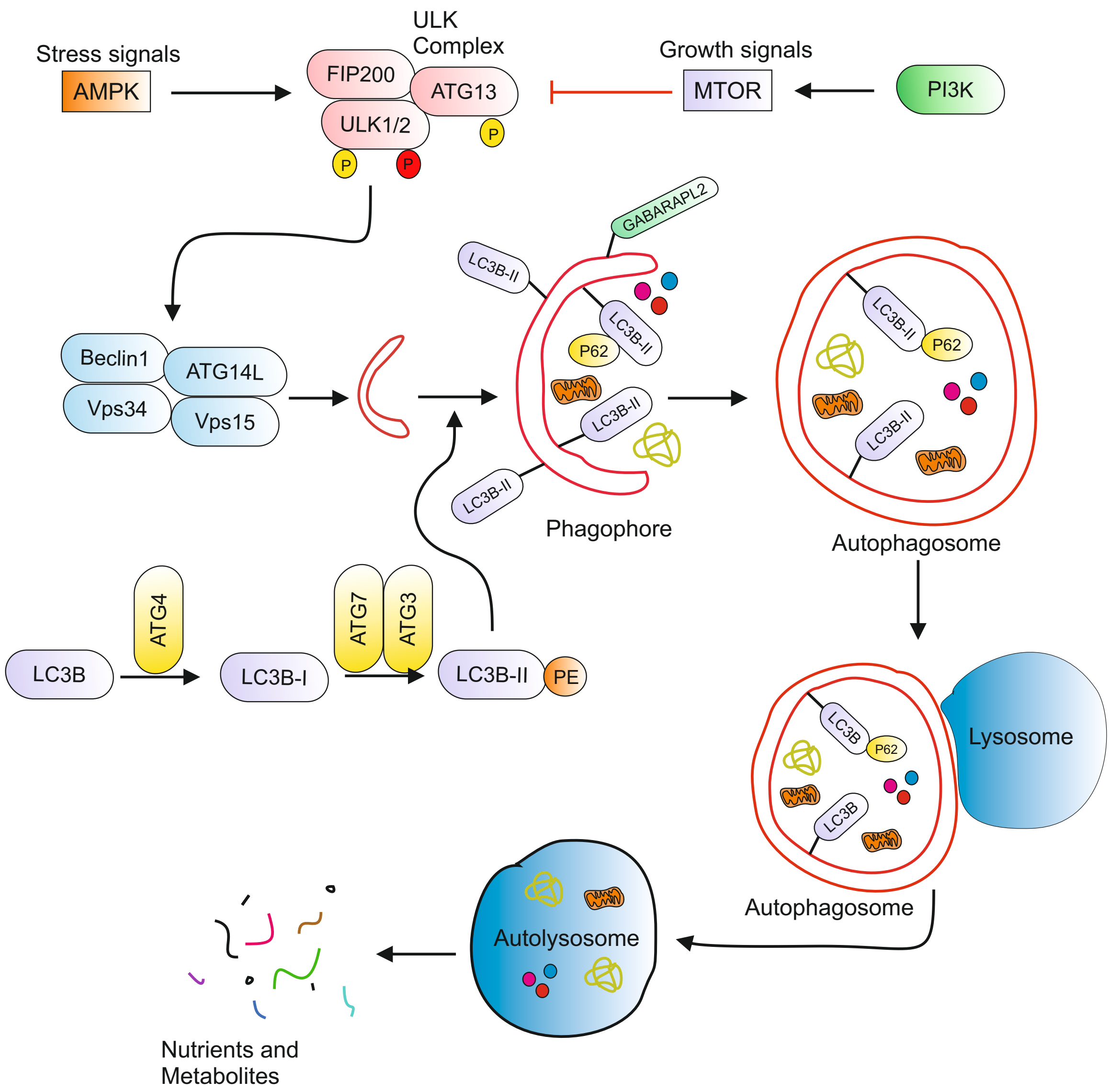

Supplementary Figure S1. The autophagy pathway. Autophagy is typically regulated by mTORC1 signaling, whereby inhibition of mTOR activation by the depletion of nutrients activates the pre-initiation complex containing ULK1/2, ATG13 and FIP200.[9] Subsequent activation of the VPS34/PIK3C3-BECN1 class-III containing complex sees the generation of phosphatidylinositol 3-phosphate at the site of autophagosome biogenesis.[10] Functional autophagy requires conjugation of phosphatidylethanolamine to LC3 (LC3A, B, C) and GABARAP (GABARAP, GABARAPL1 and GABARAPL2) family proteins, which is catalysed by ATG3 and ATG7.[11, 12] The lipid-modified form of these proteins, best studied with LC3B-II, is required for autophagosome membrane elongation, closure and autophagosome-lysosome fusion events.[13-15]

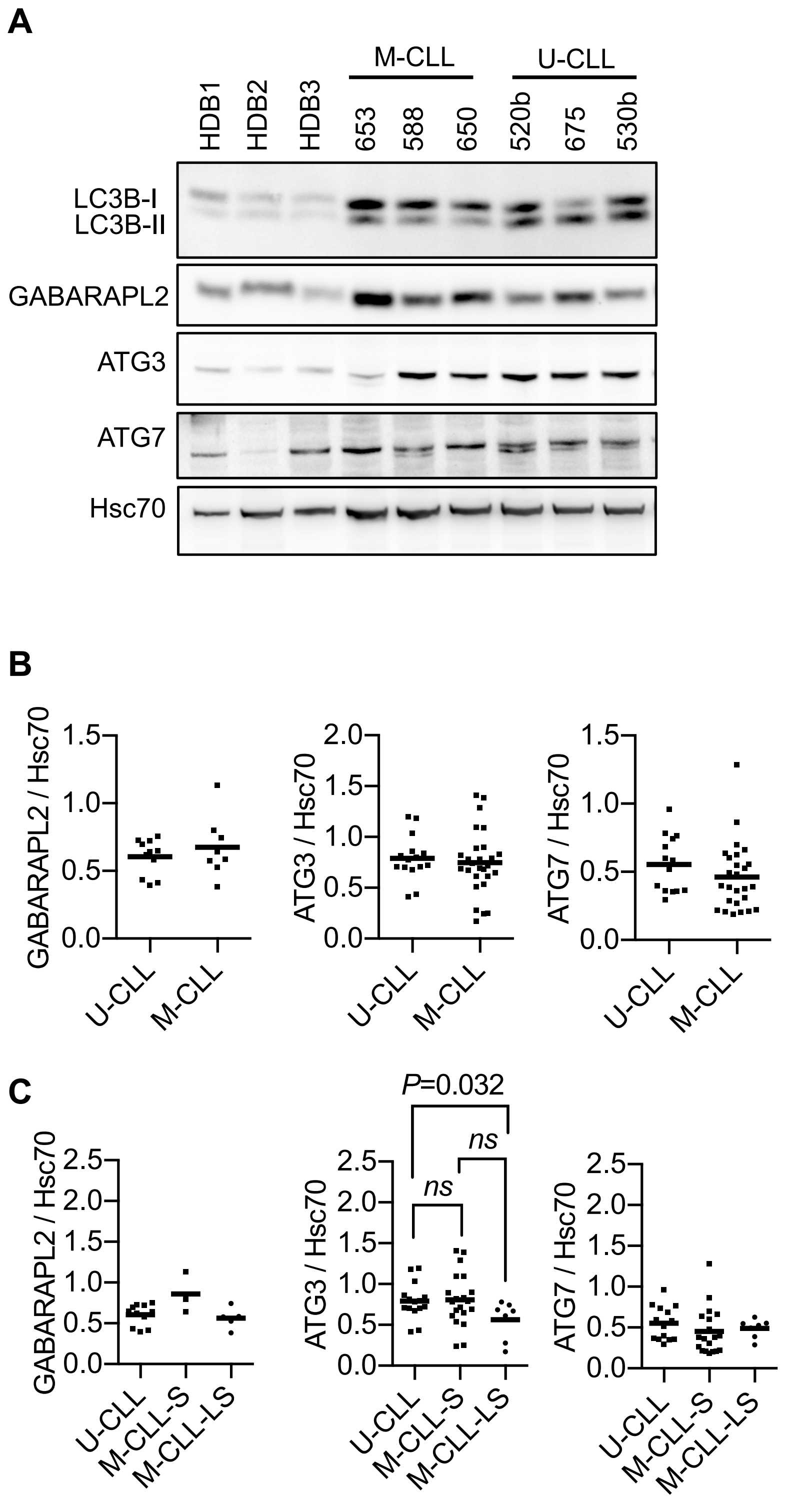

Supplementary Figure S2. Basal autophagy-protein levels in CLL. (**A**) Protein was extracted from snap-frozen PBMC's isolated from CLL patient samples or healthy donor B cells (HDB) purified by negative selection, and the levels of LC3B-II (n=43), GABARAPL2 (n=19), ATG3 (n=42) and ATG7 (n=40) evaluated by immunoblotting. Hsc70 was used as a loading control. A representative immunoblot is shown. The expression of GABARAPL2, ATG3 and ATG7 is shown characterised according to (**B**) *IGHV* status, and (**C**) anti-IgM induced iCa^{2+} flux defined by U-CLL signallers, M-CLL signallers (MCLL-S) (>5% iCa^{2+} flux) and M-CLL low signallers (M-CLL-LS) (≤5% iCa^{2+} flux). Mean values are indicated. A Mann-Whitney test was used for statistical analysis.

A

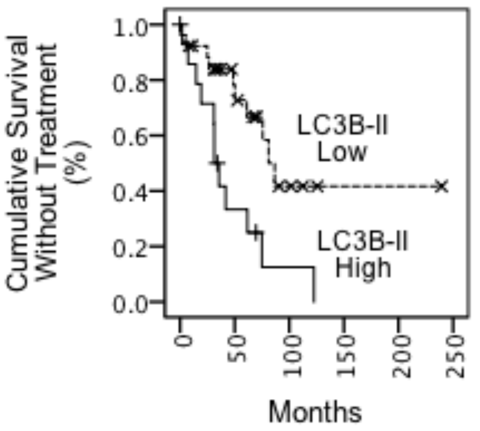

	Median Months	95% CI	Events	P-Value
LC3B-II Low	81	64-98	10/26	0.004
LC3B-II High	31	26-37	12/15	

B

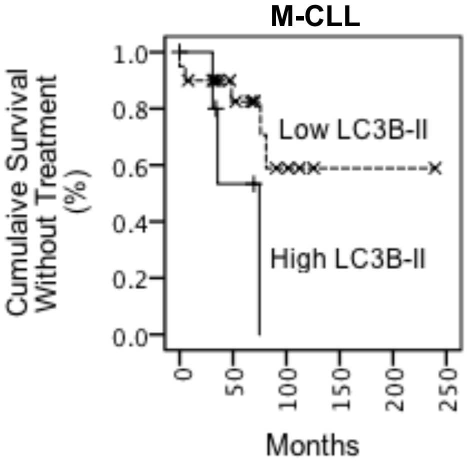

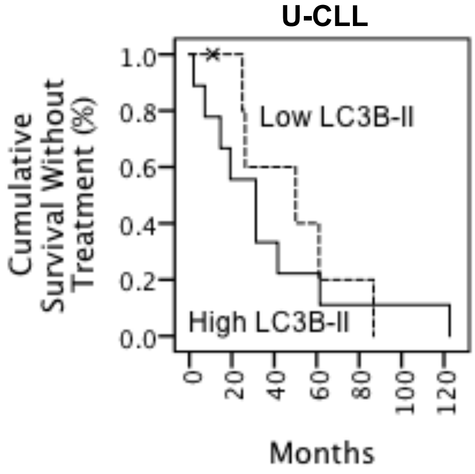

	Median Months	95% CI	Events	*P*-Value
LC3B-II Low	UNOB	UNOB	5/20	0.048
LC3B-II High	75	UNOB	3/6	

UNOB = unobtainable

	Median Months	95% CI	Events	*P*-Value
LC3B-II Low	50	0-101	5/6	0.638
LC3B-II High	31	14-48	9/9	

Supplementary Figure S3. The significance of basal LC3B-II protein levels and TTFT in CLL. (**A**) The progression of patients from first lymphocytosis to treatment (months) is shown for patients with High LC3B-II (9 U-CLL; 6 M-CLL) (solid line) or Low LC3B-II (6 U-CLL; 20 M-CLL) (dashed line). The High and Low LC3B-II cut-off was 0.71 determined by ROC analysis following protein band density quantitation. Survival analysis was performed using the Kaplan-Meier algorithm in SPSS. A Log Rank (Mantel-Cox) test was used to determine significance. (**B**) The progression of CLL patients with either high or low LC3B-II levels divided by *IGHV* mutational status.

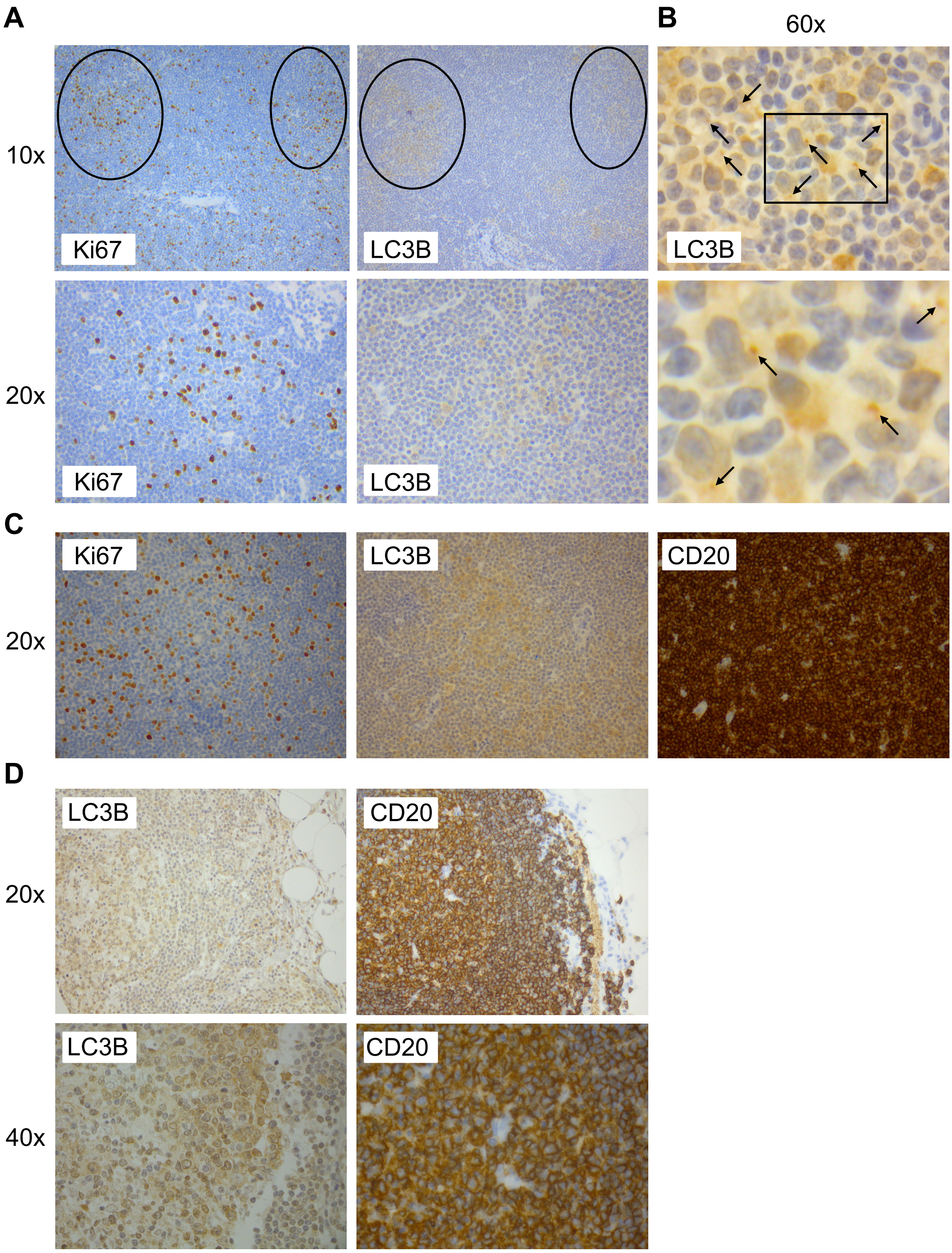

Supplementary Figure S4. Expression of LC3B within Ki67+ proliferation centres *in vivo*. (**A**) Ki67 and LC3B staining was performed on serially cut tissue sections. Results are representative of a total of 30 CLL lymph node biopsies analysed. A 10x and 20X image is shown for both markers. Matched staining of Ki67 and LC3B (10x and 20x magnification). Black circles highlight proliferation centres (PC). (**B**) Higher magnification (60X) from the sections shown in part (A). LC3B staining of autophagosomes (brown puncta) is highlighted by black arrows. The box indicates the area shown in the lower panel of (B). (**C**) Ki67, LC3B and CD20 staining was performed on sequentially cut tissue sections. (**D**) LC3B and CD20 staining of sequentially cut tissue sections from a normal reactive mesenteric lymph node.

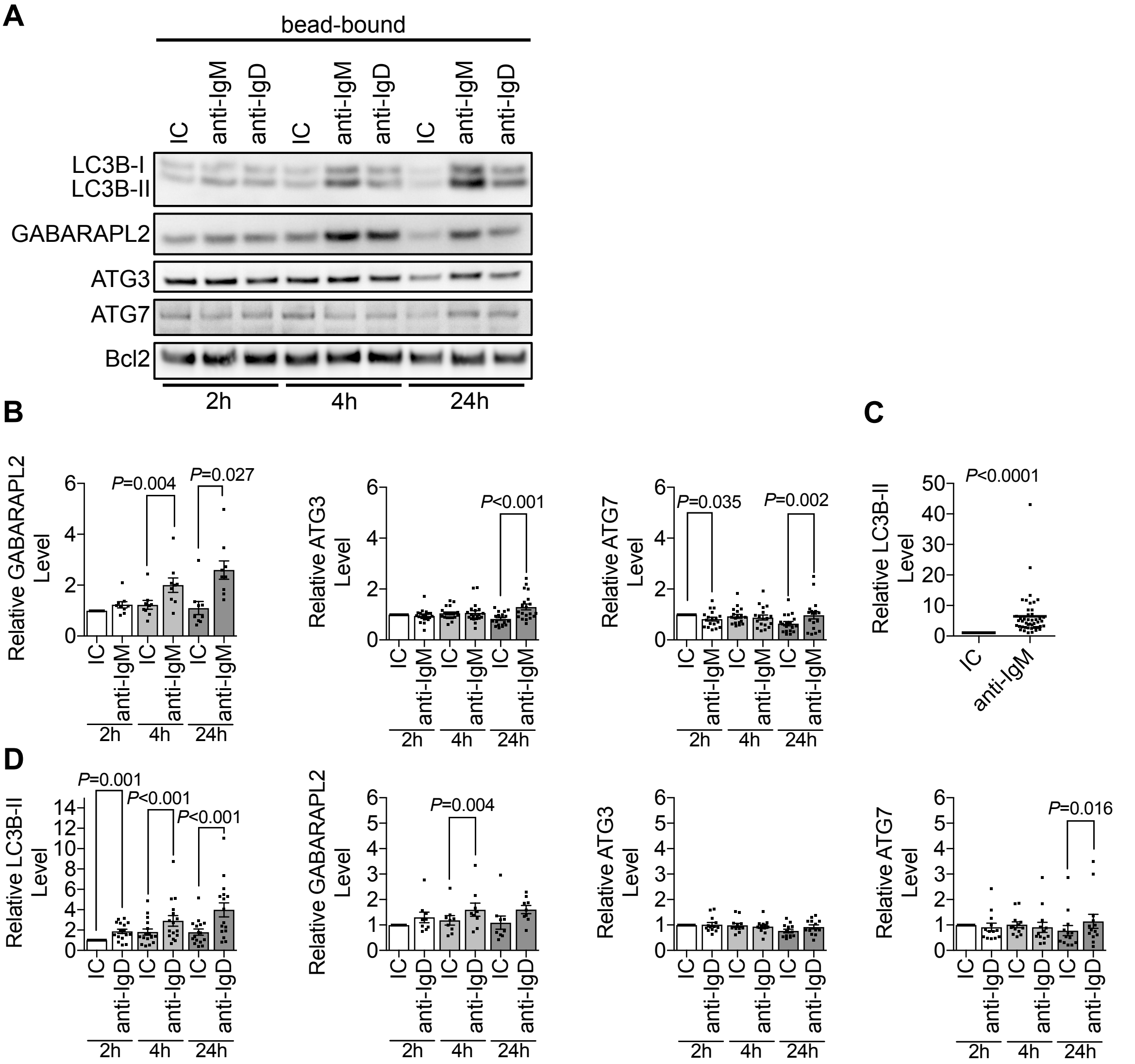

Supplementary Figure S5. Changes in autophagy-protein levels downstream of BCR-engagement in CLL. (**A**) CLL samples were treated with bead-bound isotype control antibody (IC), anti-IgM or anti-IgD for 2, 4 or 24 hours and the level of LC3B-II (IgM n=21; IgD 16), GABARAPL2 (IgM and IgD n=9), ATG3 (IgM n=20; IgD n=13) and ATG7 (IgM n=18; IgD 13) were evaluated by immunoblotting. Bcl2 was used as a loading control. A representative blot is shown. Blots were quantified and the mean fold-change (± S.E.M) in GABARAPL2, ATG3 and ATG7 with anti-IgM (**B**) or anti-IgD (**D**) at each time-point verses IC at 2 hours is shown. (**C**) A larger cohort of patient samples (n=45) were treated with bead-bound IC or anti-IgM for 24 hours and LC3B-II levels were assessed by immunoblotting. The mean fold-change in LC3B-II with anti-IgM verses IC is shown. A Wilcoxon matched-pairs signed rank test was used for statistical analysis.

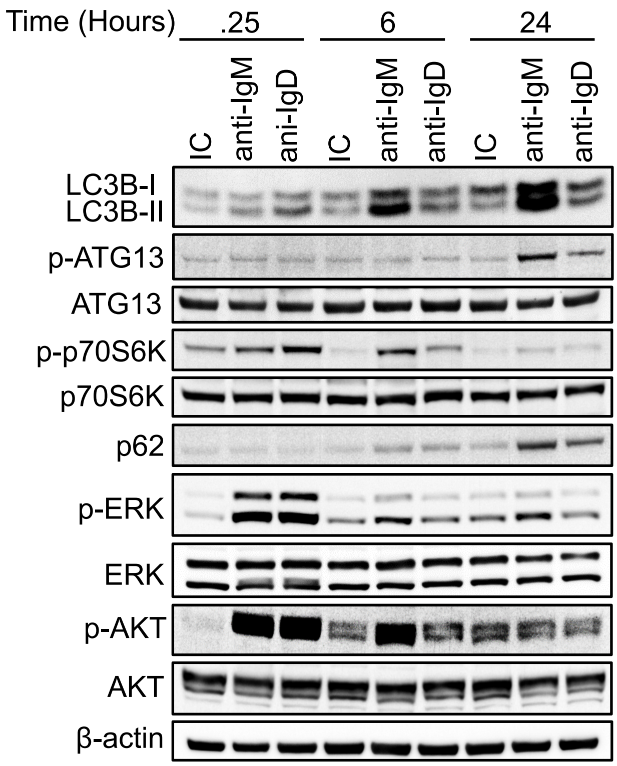

Supplementary Figure S6. Analysis of BCR and autophagy pathways. (**A**) CLL samples (n=3) were treated with bead-bound isotype control antibody (IC) or anti-IgM for time-points up to 24 hours and p62, LC3B-II, and total and phospho-ATG13, p70 S6 kinase, ERK and AKT levels assessed by immunoblotting. β-actin was used as a loading control. A representative blot is shown.

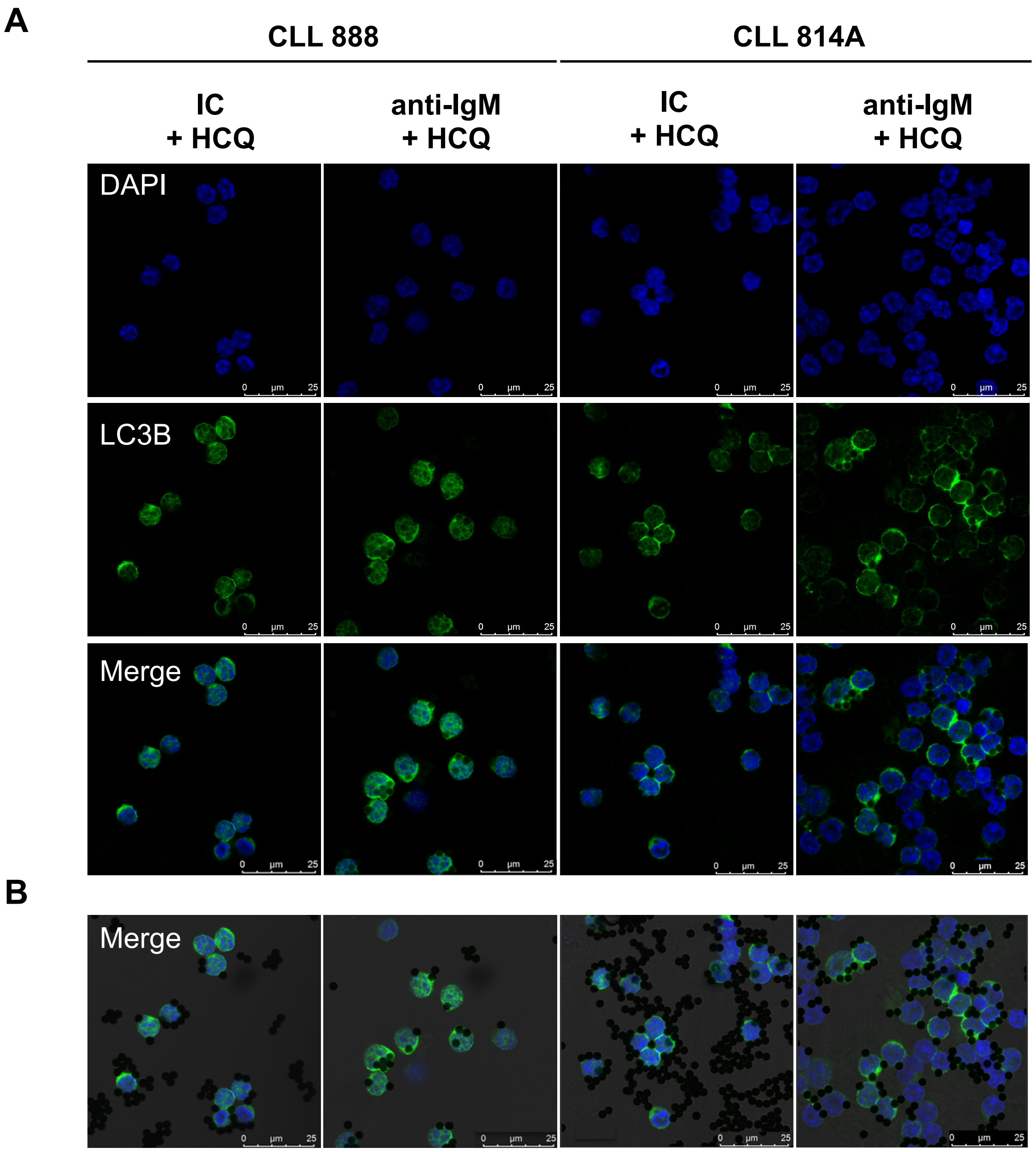

Supplementary Figure S7. The effect of BCR-engagement on autophagosome formation. CLL samples were stimulated with either bead-bound isotype control antibody (IC) or anti-IgM in the presence of HCQ for 24 hours prior to staining with LC3B (green) and DAPI nuclear stain (blue). Two representative samples (888 and 814A) are shown of a total 3 CLL samples analysed. (**A**) DAPI, LC3B and merged images, and (**B**) merged images to include the bright field image to show bead-bound IC or anti-IgM localisation.

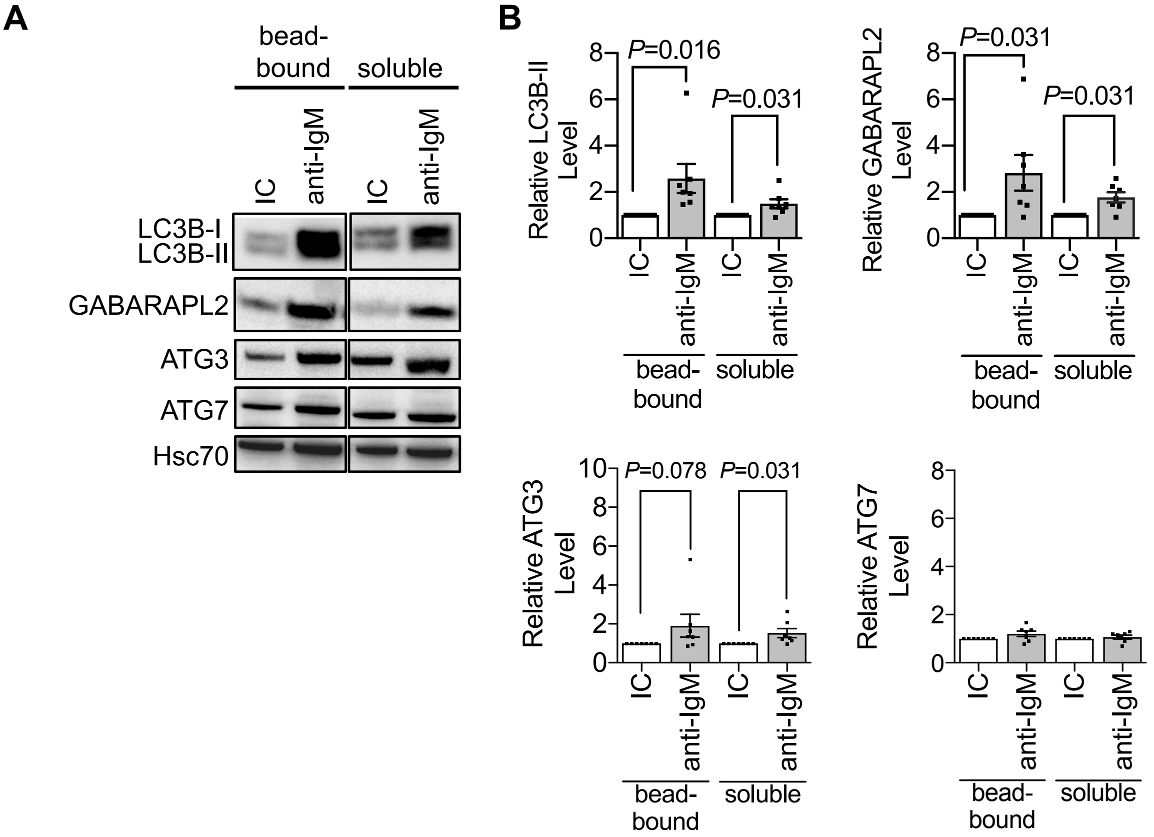

Supplementary Figure S8. Soluble and bead-bound anti-IgM both promote autophagy in CLL. (**A**) CLL samples were treated with bead-bound or soluble isotype control antibody (IC) or anti-IgM for 24 hours in the presence of HCQ before assessment of LC3B-II (n=7), GABARAPL2 (n=7), ATG3 (n=7) and ATG7 (n=7) levels by immunoblotting. Hsc70 was used as a loading control. A representative immunoblot is shown. (**B**) Blots were quantified and the mean fold change (± S.E.M) in the level of each protein with anti-IgM versus bead-bound or soluble IC is shown. Mean values are indicated. A Wilcoxon matched-pairs signed rank test was used for statistical analysis.

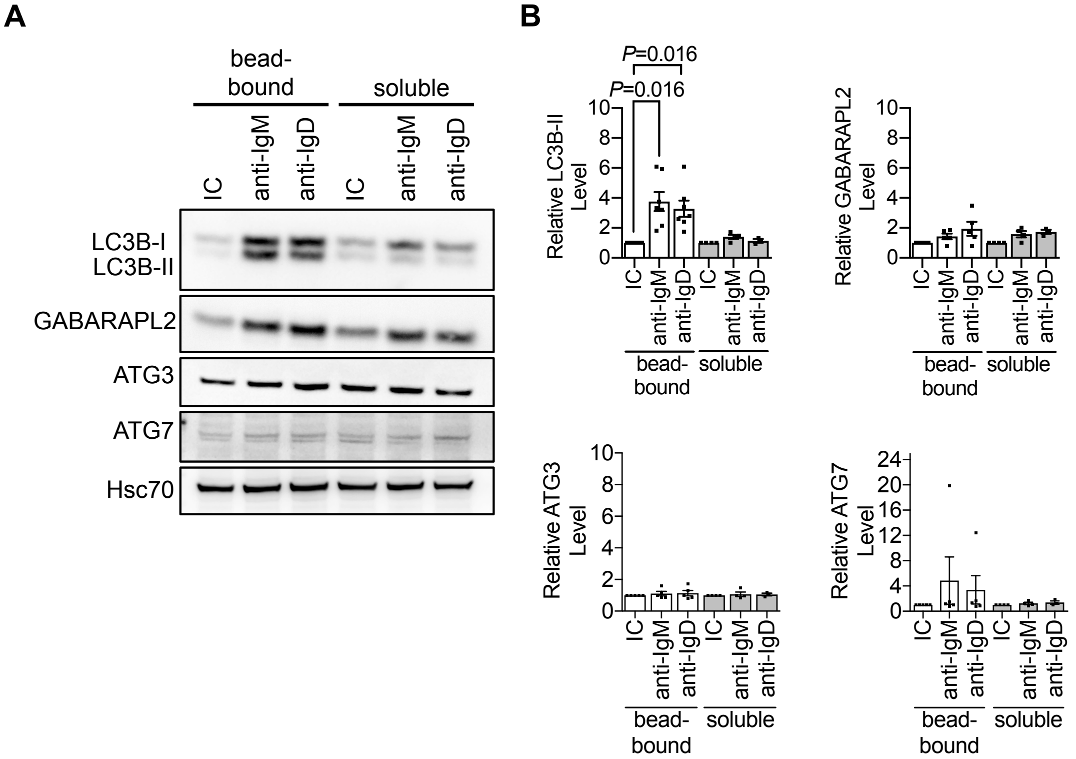

Supplementary Figure S9. BCR-engagement increases autophagy-marker protein levels in HDB. (**A**) Fresh B-cells were purified by negative selection from healthy donor PBMCs and stimulated with either bead-bound or soluble isotype control antibody (IC), anti-IgM or anti-IgD in the presence of HCQ for 24 hours. The level of LC3B-II (n=7), GABARAPL2 (n=5), ATG3 (n=5) and ATG7 (n=5) were evaluated by immunoblotting. A representative immunoblot is shown. Hsc70 was used as a loading control. (**B**) Blots were quantified and the mean fold change (± S.E.M) in protein levels with each treatment versus bead-bound or soluble IC is shown. Mean values are indicated. A Wilcoxon matched-pairs signed rank test was used for statistical analysis.

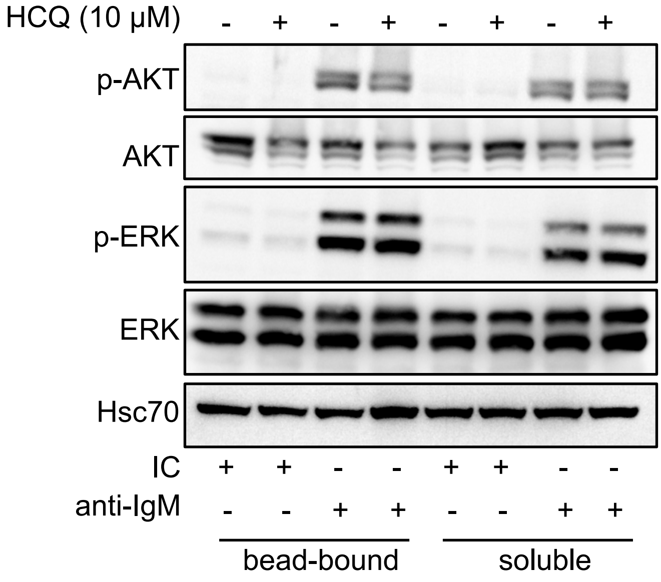

Supplementary Figure S10. The effect of HCQ on anti-IgM-mediated BCR signaling and LC3B-II levels. CLL samples were treated with or without HCQ for 1 hour prior to treatment with soluble or bead-bound isotype control antibody (IC) or anti-IgM for 30 minutes. Phosphorylated and total AKT and ERK levels were assessed by immunoblotting. Hsc70 was used as a loading control. A representative blot is shown (n=2).

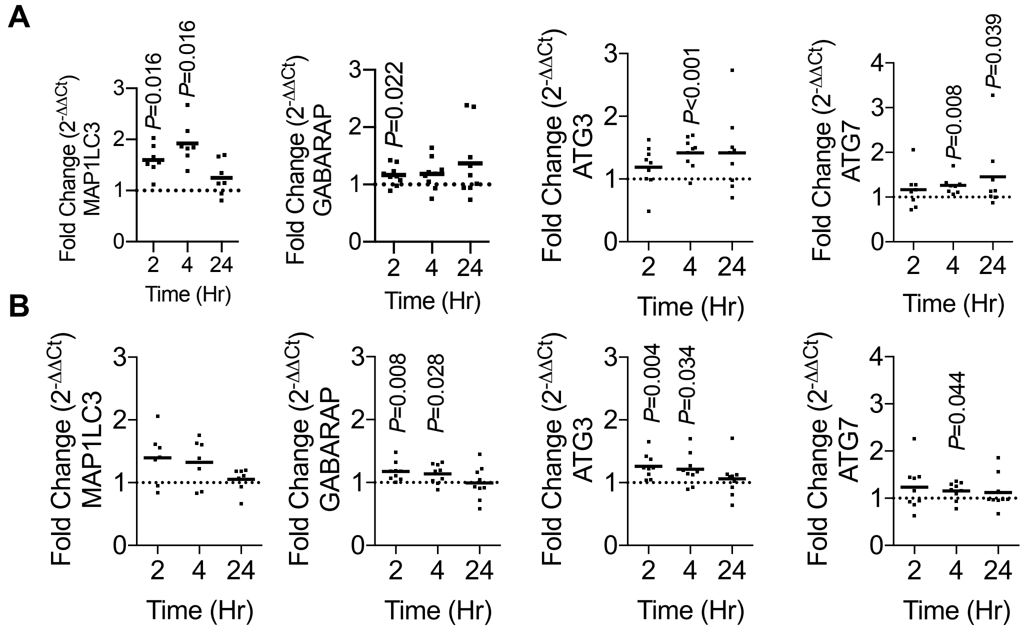

Supplementary Figure S11. BCR-engagement promotes autophagy gene expression. CLL samples were treated with bead-bound isotype control antibody (IC), anti-IgM (**A**) or anti-IgD (**B**) for 2, 4 or 24 hours and the expression of MAP1LC3 (n=7), GABARAP (n=9), ATG3 (n=9) and ATG7 (n=8 and n=9 for anti-IgM and anti-IgD respectively) mRNA assessed by qPCR. β2 microglobulin was used to normalise gene expression. The mean fold-change (± S.E.M) in the expression of each gene following anti-IgM or anti-IgD treatment at each time-point compared to IC is shown. Mean values are indicated. Either a t-test or Wilcoxon matched-pairs signed rank test was used for statistical analysis.

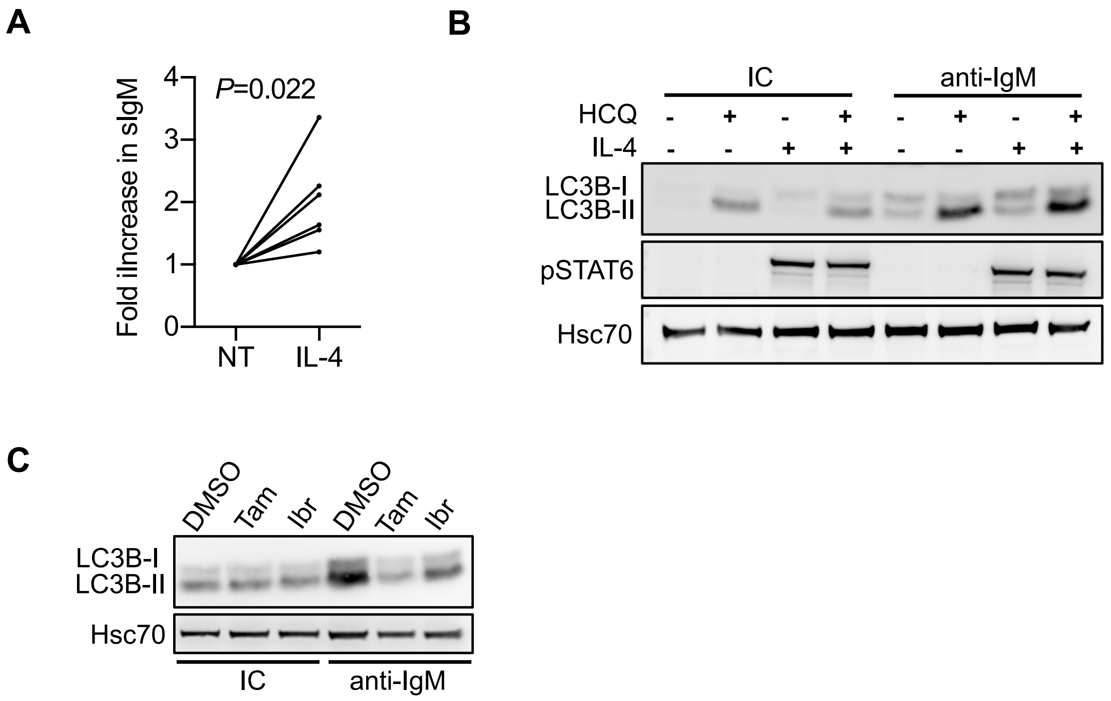

Supplementary Figure S12. BCR-mediated autophagy is regulated by BCR signaling. (A) CLL samples (n=6) were treated with or without IL-4 (10ng/ml) for 24 hours before sIgM expression was assessed by flow cytometry. The fold-change in sIgM expression following IL-4 treatment compared no treatment (NT) is shown. A t-test was used for used for statistical analysis. (**B**) CLL samples (n=6) were treated with or without IL-4 (10ng/ml) and/or HCQ for 24 hours prior to treatment with bead-bound isotype control antibody (IC) or anti-IgM for 24 hours. Phospho-STAT6 and LC3B-II levels were evaluated by immunoblotting. Hsc70 was used as a loading control. A representative immunoblot is shown. (**C**) CLL samples (n=10) were treated with HCQ and a SYK (tamatinib; Tam) or BTK (ibrutinib; Ibr) inhibitor (both 5µM) for 1 hour prior to stimulation with bead-bound IC or anti-IgM for 24 hours and the LC3B-II level evaluated by immunoblotting. Hsc70 was used as a loading control. A representative immunoblot is shown.

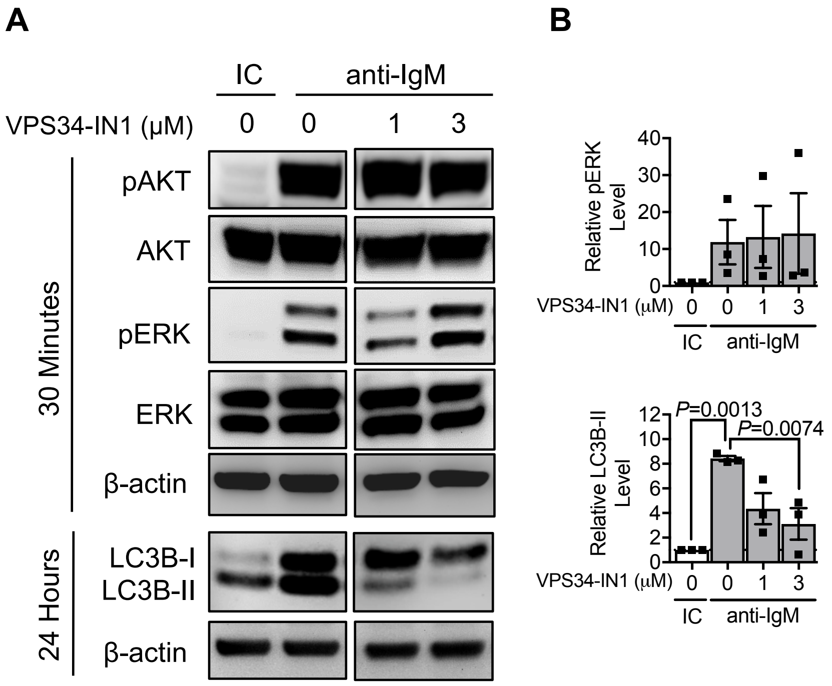

Supplementary Figure S13. VPS34-IN1 does not inhibit anti-IgM-mediated BCR signaling. (**A**) CLL samples (n=3) were pre-treated with 3µM VPS34-IN1 for 1 hour, prior to addition of bead-bound anti-IgM or isotype control antibody (IC) for 30 minutes or 24 hours. The level of total and phospho-AKT (pAKT473) and ERK, and LC3B-II were assessed by immunoblotting to examine the effect of VPS34-IN1 on anti-IgM-mediated signaling and autophagy respectively. Caspase inhibitor QVD-O-Ph was included from the start of the experiment to inhibit caspase-dependant cell death. β-actin was used as a loading control. A representative blot is shown. (**B**) Blots were quantified and the mean fold-change (± S.E.M) in protein levels with each treatment versus IC is shown in graph. A Two-Way ANOVA was used for statistical analysis.

Supplementary References

1. Hallek M, Cheson BD, Catovsky D, Caligaris-Cappio F, Dighiero G, Dohner H*, et al.* Guidelines for the diagnosis and treatment of chronic lymphocytic leukemia: a report from the International Workshop on Chronic Lymphocytic Leukemia updating the National Cancer Institute-Working Group 1996 guidelines. *Blood* 2008 Jun 15; **111**(12)**:** 5446-5456.

2. Mockridge CI, Potter KN, Wheatley I, Neville LA, Packham G, Stevenson FK. Reversible anergy of sIgM-mediated signaling in the two subsets of CLL defined by VH-gene mutational status. *Blood* 2007 May 15; **109**(10)**:** 4424-4431.

3. Lanham S, Hamblin T, Oscier D, Ibbotson R, Stevenson F, Packham G. Differential signaling via surface IgM is associated with VH gene mutational status and CD38 expression in chronic lymphocytic leukemia. *Blood* 2003 Feb 1; **101**(3)**:** 1087-1093.

4. Steele AJ, Prentice AG, Cwynarski K, Hoffbrand AV, Hart SM, Lowdell MW*, et al.* The JAK3-selective inhibitor PF-956980 reverses the resistance to cytotoxic agents induced by interleukin-4 treatment of chronic lymphocytic leukemia cells: potential for reversal of cytoprotection by the microenvironment. *Blood* 2010 Nov 25; **116**(22)**:** 4569-4577.

5. Steele AJ, Prentice AG, Hoffbrand AV, Yogashangary BC, Hart SM, Nacheva EP*, et al.* p53-mediated apoptosis of CLL cells: evidence for a transcription-independent mechanism. *Blood* 2008 Nov 01; **112**(9)**:** 3827-3834.

6. McCarthy A, Marzec J, Clear A, Petty RD, Coutinho R, Matthews J*, et al.* Dysregulation of autophagy in human follicular lymphoma is independent of overexpression of BCL-2. *Oncotarget* 2014 Nov 30; **5**(22)**:** 11653-11668.

7. Chen R, Guo L, Chen Y, Jiang Y, Wierda WG, Plunkett W. Homoharringtonine reduced Mcl-1 expression and induced apoptosis in chronic lymphocytic leukemia. *Blood* 2011 Jan 6; **117**(1)**:** 156-164.

8. Chou TC, Talalay P. Quantitative analysis of dose-effect relationships: the combined effects of multiple drugs or enzyme inhibitors. *Adv Enzyme Regul* 1984; **22:** 27-55.

9. Kim J, Kundu M, Viollet B, Guan KL. AMPK and mTOR regulate autophagy through direct phosphorylation of Ulk1. *Nat Cell Biol* 2011 Feb; **13**(2)**:** 132-141.

10. Axe EL, Walker SA, Manifava M, Chandra P, Roderick HL, Habermann A*, et al.* Autophagosome formation from membrane compartments enriched in phosphatidylinositol 3-phosphate and dynamically connected to the endoplasmic reticulum. *J Cell Biol* 2008 Aug 25; **182**(4)**:** 685-701.

11. Ichimura Y, Kirisako T, Takao T, Satomi Y, Shimonishi Y, Ishihara N, *et al.* A ubiquitin-like system mediates protein lipidation. *Nature* 2000 Nov 23; **408**(6811)**:** 488-492.

12. Kabeya Y, Mizushima N, Yamamoto A, Oshitani-Okamoto S, Ohsumi Y, Yoshimori T. LC3, GABARAP and GATE16 localize to autophagosomal membrane depending on form-II formation. *J Cell Sci* 2004 Jun 01; **117**(Pt 13)**:** 2805-2812.

13. Weidberg H, Shpilka T, Shvets E, Abada A, Shimron F, Elazar Z. LC3 and GATE-16 N termini mediate membrane fusion processes required for autophagosome biogenesis. *Dev Cell* 2011 Apr 19; **20**(4)**:** 444-454.

14. Weidberg H, Shvets E, Shpilka T, Shimron F, Shinder V, Elazar Z. LC3 and GATE-16/GABARAP subfamilies are both essential yet act differently in autophagosome biogenesis. *EMBO J* 2010 Jun 02; **29**(11)**:** 1792-1802.

15. Schaaf MB, Keulers TG, Vooijs MA, Rouschop KM. LC3/GABARAP family proteins: autophagy-(un)related functions. *FASEB J* 2016 Sep 6.
